# Assessment of deep learning assistance for the pathological diagnosis of gastric cancer

**DOI:** 10.1038/s41379-022-01073-z

**Published:** 2022-04-08

**Authors:** Wei Ba, Shuhao Wang, Meixia Shang, Ziyan Zhang, Huan Wu, Chunkai Yu, Ranran Xing, Wenjuan Wang, Lang Wang, Cancheng Liu, Huaiyin Shi, Zhigang Song

**Affiliations:** 1 Department of Pathology, Chinese PLA General Hospital, 100853 Beijing, China; 2 Thorough Images, 100176 Beijing, China; 3 Institute for Interdisciplinary Information Sciences, Tsinghua University, 100084 Beijing, China; 4 Department of Biostatistics, Peking University First Hospital, 100102 Beijing, China; 5 Department of Dermatology, Affiliated Hospital of North China University of Science and Technology, 063000 Tangshan, China; 6 Medical Big Data Center, Chinese PLA General Hospital, 100853 Beijing, China; 7 Department of Pathology, Beijing Shijitan Hospital, Capital Medical University, 100038 Beijing, China; 8 Chinese Academy of Inspection and Quarantine, 100176 Beijing, China; 9 Department of Dermatology, Chinese PLA General Hospital, 100853 Beijing, China

**Keywords:** Gastric cancer, Translational research, Cancer screening, Pathology

## Abstract

Previous studies on deep learning (DL) applications in pathology have focused on pathologist-versus-algorithm comparisons. However, DL will not replace the breadth and contextual knowledge of pathologists; rather, only through their combination may the benefits of DL be achieved. A fully crossed multireader multicase study was conducted to evaluate DL assistance with pathologists’ diagnosis of gastric cancer. A total of 110 whole-slide images (WSI) (50 malignant and 60 benign) were interpreted by 16 board-certified pathologists with or without DL assistance, with a washout period between sessions. DL-assisted pathologists achieved a higher area under receiver operating characteristic curve (ROC-AUC) (0.911 vs. 0.863, *P* = 0.003) than unassisted in interpreting the 110 WSIs. Pathologists with DL assistance demonstrated higher sensitivity in detection of gastric cancer than without (90.63% vs. 82.75%, *P* = 0.010). No significant difference was observed in specificity with or without deep learning assistance (78.23% vs. 79.90%, *P* = 0.468). The average review time per WSI was shortened with DL assistance than without (22.68 vs. 26.37 second, *P* = 0.033). Our results demonstrated that DL assistance indeed improved pathologists’ accuracy and efficiency in gastric cancer diagnosis and further boosted the acceptance of this new technique.

## Introduction

Gastric cancer is the third most common cause of cancer-related death worldwide, and it ranks second in China^1–3^. It is estimated that over 1 million new cases of gastric cancer are diagnosed annually globally^3^. Histopathological evaluation of gastric specimens is essential for clinical management, which requires experienced pathologists and is time-consuming. However, a shortage of pathologists exists globally. It has been reported that there is a shortage of 90,000 pathologists in China, and the deficiency is more severe in many African countries^4,5^. Western countries are also facing a similar problem due to the increasing retirement of pathologists^6,7^.

Artificial intelligence, especially deep learning algorithm, has shown better or on par performance with human pathologists in several fields, using hematoxylin and eosin (H&E)-stained whole-slide images (WSIs)^8–10^. Ehteshami et al.^8^ demonstrated that deep learning achieved better performance than a panel of 11 pathologists in the detection of lymph node metastasis of breast cancer. Recent studies have shown that deep learning achieved relatively high sensitivity and specificity in diagnosing gastrointestinal cancer^11^, lung cancer^12^, prostate cancer^13,14^, and others^15,16^.

We have developed a deep learning algorithm for gastric cancer detection, and it achieved a sensitivity near 100% and a specificity of 80.6% in 3212 real-world WSIs scanned by different scanners^17^. In an internal examination, the performance of the algorithm was on par with 12 pathologists in interpreting 100 WSIs^17^. However, our study and previous studies focused on pathologist-versus-algorithm comparisons rather than their combination^8,9,17^. An accurate deep learning algorithm will not replace the breadth and contextual knowledge of pathologists. Rather, only through their integration into a clinical setting may the benefits of the algorithm be fully achieved^18^. Based on the above considerations, we conducted a reader study to evaluate the performance of pathologists in interpreting WSIs of gastric specimens with and without deep learning assistance.

## Materials and methods

### Cases enrollment

A total of 110 gastric slides based on pathology reports were retrospectively selected from PLA general hospital (PLAGH) between 1 July 2019 and 31 December 2020. Among these specimens, 60 were benign and 50 were malignant, which basically represented all gastric specimens encountered in the daily workflow (Table 1). The 110 gastric slides were from 110 distinct cases. All samples were biopsy specimens because surgical specimens often indicate malignant tumors, which may affect pathologists’ judgment.

**Table 1:** Test set for assessment study.

Gastric specimen	No. WSIs
Benign (non-gastric cancer)	60
Low-grade intraepithelial neoplasia	6
Other benign lesions or normal mucosa	54
Malignant (gastric cancer)	50
Well-differentiated adenocarcinoma (including high-grade intraepithelial dysplasia)	18
Moderated-differentiated adenocarcinoma	10
Poorly-differentiated adenocarcinoma	11
Mucinous adenocarcinoma	2
Poorly cohesive adenocarcinoma including signet ring cell and other subtypes	9

*WSI* whole-slide pathological images.

### Reference standard diagnosis

The reference gold standard diagnosis was established for each of the 110 slides. Three senior pathologists from PLAGH independently reviewed the glass slides and made a diagnosis for each case. For cases with inconsistent opinions, all three specialists reviewed the slides, including immunohistochemistry, together using a multiheaded microscope to reach a consensus. Slides were scanned into WSIs with a KF-PRO-005 scanner (0.238 μm × 0.238 μm per pixel). The resulting WSIs were inspected one by one to ensure image quality. WSIs with out-of-focus or missing tissue were rescanned.

### Pathologists

A total of 16 board-certified anatomic pathologists from 12 different hospitals participated in this study. They were not participants in either the test set enrollment or establishing of reference standard diagnoses. Their anatomic pathology experience ranged from 6 to 20 years. Because most pathologists did not have experience of reviewing WSIs with or without deep learning assistance, all of them read no <50 WSIs to establish familiarity with the reading system within a month prior to the assessment study. All pathologists participated voluntarily, and understood and agreed with the basic principles and purposes of this research.

### Deep learning algorithm

In our previous work^17^, we utilized a convolutional neural network of DeepLab v3 architecture for gastric cancer detection. The deep learning algorithm was trained with 2123 pixel-level annotated H&E-stained WSIs and achieved a sensitivity of 99.6% with an average specificity of 80.6% on a real-world test dataset of 3212 WSIs, digitalized by three scanners. The generalization ability of the algorithm was further tested with 1582 WSIs from 2 other medical centers. The deep learning algorithm can automatically output pixel-level malignant probabilities, which were integrated into the slide-level prediction.

### Study design

**Fig. 1:**
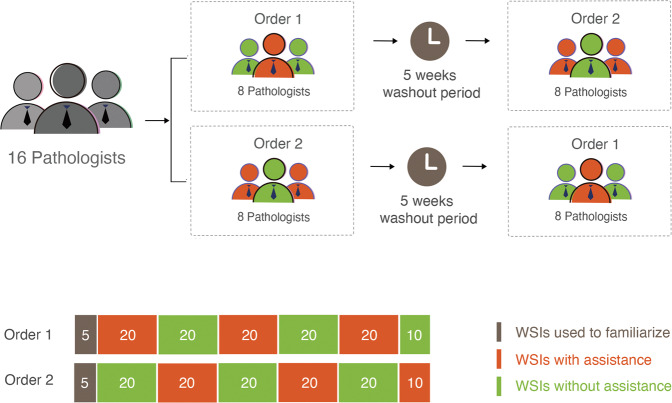
Study design. The 16 pathologists reviewed the same WSIs in the same sequence but with different modalities: with or without deep learning assistance. The 16 pathologists were randomized into two assistance “orders.” Each rectangle indicates a set of WSIs; the color of the rectangle indicates the modality, and the number in the rectangle indicates the number of WSIs. The pathologists reviewed 5 images (not part of the test set) for familiarity and a total of 110 images for formal review.

A fully crossed multireader multicase (MRMC) study was performed to evaluate deep learning assistance in pathologists’ diagnosis of gastric lesions. A total of 110 WSIs (50 malignant and 60 benign) were interpreted by 16 board-certified anatomic pathologists with or without deep learning assistance, separated by a washout period of 5 weeks (Fig. 1). To mitigate bias for possible performance differences at the beginning versus the end of interpreting the test set, the 110 WSIs were divided into blocks of 20 WSIs (the last block with 10 WSIs), with each block containing roughly the same proportion of benign and malignant WSIs, but in random order. In addition, to establish familiarity for reviewing WSIs, each order began with a review of 5 WSIs. The 16 pathologists were randomized into 2 groups, either of which began with (order 1) or without (order 2) deep learning assistance first. In either order, the WSIs interpreted were identical; the only difference was with or without deep learning assistance.

### Sample size

We calculated the sample size using the “Multi- and Single-Reader Sample Size Program for Diagnostic Studies” (available at https://perception.lab.uiowa.edu/power-sample-size-estimation), which is based upon the methods of Hillis, Obuchowski, and Berbaum. A pilot study demonstrated 16 readers and 100 WSIs would provide more than 90% power with a 5% significance level, with the aim of proving the superiority of the area under receiver operating characteristic curve (ROC-AUC) of the pathologists with assistance over without assistance.

### With or without deep learning assistance review

In the assessment study, when the pathologists reviewed the WSIs, modalities (with or without deep learning assistance) switched every 20 WSI intervals. For WSIs with deep learning assistance, a heat-map flagging suspicious malignant areas over the WSI could be turned on and off by tapping the space bar on the keyboard. For WSIs without assistance, only the WSI was displayed. The participants provided a diagnosis by clicking the buttons on the screen (Supplementary Fig. S1). WSIs were presented on a 13.3″ 2560*1600 LED monitor (Apple MacBook Pro 13.3).

### WSI review timing

To simulate the clinical workflow as much as possible, 16 pathologists were instructed to evaluate 110 WSIs with a self-controlled pace. For each WSI, the time from opening the WSI in the viewer to final diagnosis was recorded by a background program. The pathologist could take a break during the test, and that time was not counted.

### Statistical analysis

Pathologists were requested to provide one of four different diagnoses to each WSI (malignant/ possibly malignant/ possibly benign/ benign), corresponding to a “suspicion score” from 1 to 4, which were used for building the ROCs. We analyzed the average AUC based on the readers’ suspicion score as a statistically efficient approach to evaluate the cancer and non-cancer performance metrics combined into a single measurement. These analyses were performed according to the method of Obuchowski & Rockette with Hillis adjustment to the degrees of freedom with mixed-effects models. Models were generated with pathologists, WSIs treated as random effects and the assistance modality and session (order 1 or order 2) treated as fixed effects. The trapezoidal/Wilcoxon method for curve fitting and jackknifing for the covariance estimation were used in the analysis. To compare the sensitivity and specificity between two sessions (with and without deep learning assistance), a binary-version MRMC analysis was implemented to yield a *P*-value. The average review time of each WSI was calculated for each pathologist in each session, and the paired *t*-test was used to yield the *P*-value for the difference between the two sessions. All other statistical analyses were performed in the statistical computing environment R 4.0 and SAS 9.4. No statistical adjustments were made for multiple analyses.

## Results

### Performance of pathologists with or without assistance

**Fig. 2:**
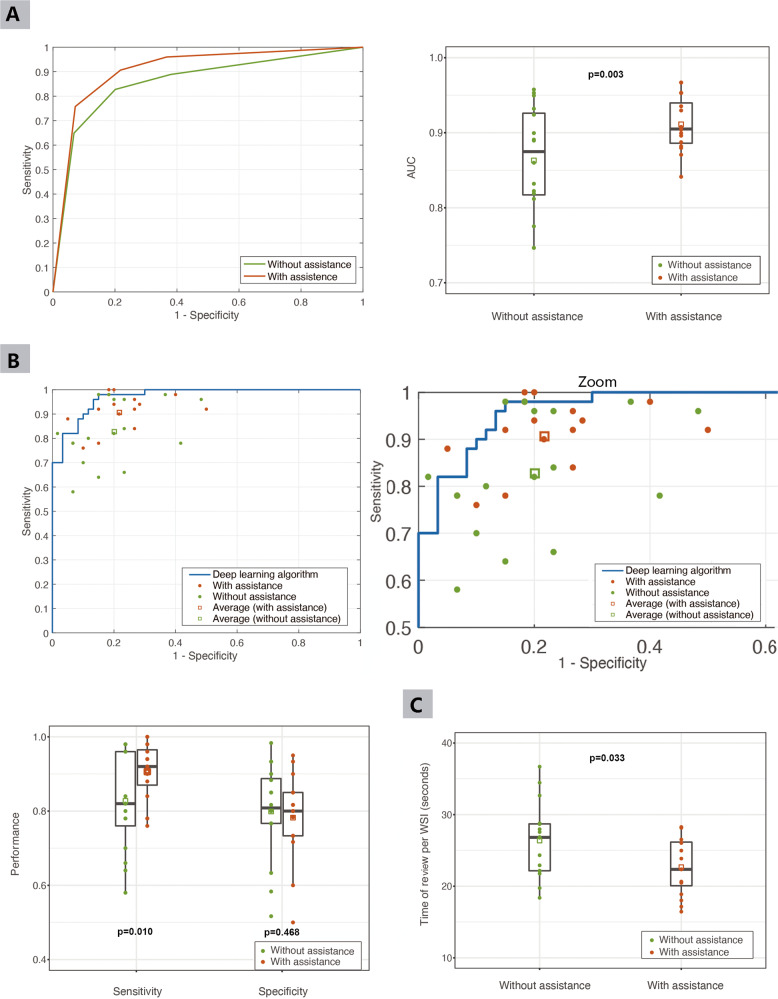
Performance of pathologists with or without deep learning assistance. **A** The average AUC of pathologists with deep learning assistance was larger than that of without (0.911 vs. 0.863, *P* = 0.003). **B** The sensitivity of the pathologists was improved with deep learning assistance compared to those without assistance (90.63% vs. 82.75%, *P* = 0.010). There was no significant difference in specificity with or without deep learning assistance (78.23% vs. 79.90%, *P* = 0.468). **C** The average review time per WSI was reduced with deep learning assistance compared to without (22.68 s vs. 26.37 s, *P* = 0.033). The circles represent the value of each pathologist, the squares indicate the average of pathologists in that modality, and the vertical lines of the box represent quartiles. AUC area under the receiver operating characteristic curve; time of review per WSI is described as the mean ± SD (standard deviation).

The pathologists marked each WSI as either malignant, possibly malignant, possibly benign or benign (Supplementary Fig. S1). The results were fitted into a ROC for each pathologist with or without deep learning assistance (trapezoidal/Wilcoxon method). The performance of the pathologists was evaluated by the ROC-AUC. The average AUCs of the pathologists with and without deep learning assistance were 0.911 and 0.863 (*P* = 0.003, 95% confidence interval [CI]: 0.018–0.079) (Fig. 2A and Table 2), which demonstrated that deep learning assistance indeed improved the diagnostic performance of the pathologists. The AUC of each pathologist with or without assistance was presented in Supplementary Table S1.

**Table 2:** Performance of pathologists with or without deep learning assistance.

Evaluation index	With assistance	Without assistance	95% CI	*P* value
AUC	0.9112	0.8631	0.0176, 0.0786	0.003
Sensitivity	0.9063	0.8275	0.0209, 0.1366	0.010
Specificity	0.7823	0.7990	−0.0637, 0.0304	0.468
Time of review per WSI (mean ± SD, seconds)	22.68 ± 4.03	26.37 ± 5.22	–	0.033

*AUC* area under the receiver operating characteristic curve, *SD* standard deviation, *95% CI* confidence interval.

According to the pathologist’s diagnosis, malignant and possibly malignant were clustered as gastric cancer, and benign and possibly benign were clustered as non-cancer. On the binary classification level, the mean sensitivities of the pathologists without and with deep learning assistance were 82.75% and 90.63% (*P* = 0.010, 95% CI: 2.09–13.66%). The mean specificities of the pathologists without and with deep learning assistance were 79.90% and 78.23% (*P* = 0.468, 95% CI: −6.37–3.04%) (Fig. 2B). A summary of the above results were shown in Table 2, and each pathologist’s sensitivity and specificity were showed in Supplementary Tables S2 and S3.

**Fig. 3:**
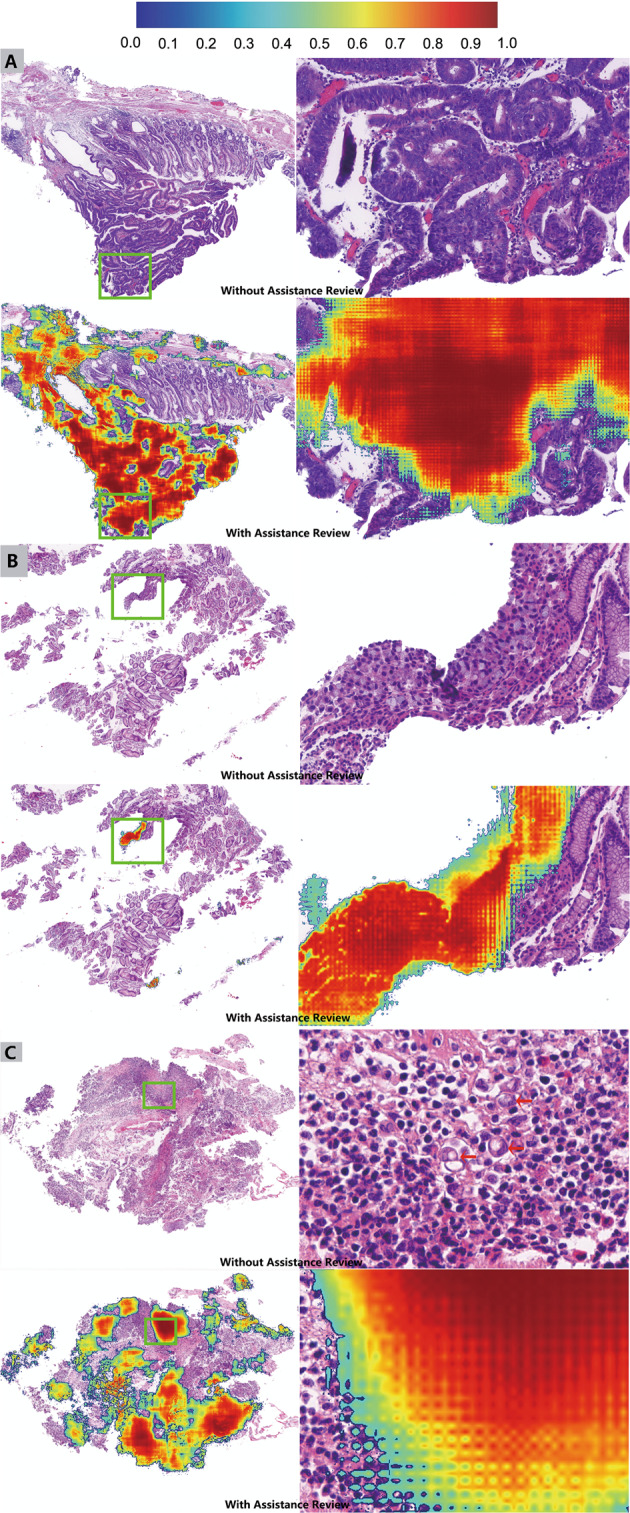
Three representative examples showing accuracy improvement after deep learning assistance. In each example, the top two cells (low power view and zoomed area of the green rectangle) represent WSIs without assistance, while the bottom represent the same WSI with assistance. **A** The gold standard diagnosis of the case is high-grade intraepithelial neoplasia. Four of 16 pathologists were uncertain about the case and misdiagnosed it as possibly benign. After deep learning flagging the suspicious areas, the accuracy of the pathologists increased from 75% to 100%. **B** Due to the very small proportion of signet ring cell carcinoma in the WSI, pathologists may miss malignant areas. After deep learning flagging suspicious areas, the accuracy increased from 62.5% to 93.75%. **C** The scattered signet ring cells (red arrow) are mixed with lymphocytes and histiocytes, making diagnosis difficult. After using deep learning assistance, the accuracy increased from 37.5% to 87.5%. The colored scale bar (top) indicates the probability for each pixel to be malignant.

We further analyzed the change in accuracy for each WSI between different assistance modalities. For cases with little difficulty, deep learning had a limited effect on the accuracy improvement, while for cases with uncertain diagnoses or small malignant areas that could be easily missed, deep learning could significantly improve the accuracy. Figure 3 shows three representative examples in which the accuracy of pathologists was significantly improved after deep learning assistance. As shown in Fig. 3A, which was a gastric high-grade intraepithelial neoplasia, 4 out of 16 pathologists diagnosed it as possibly benign. After the deep learning algorithm highlighting suspected malignant areas, the 4 pathologists changed their diagnosis to possibly malignant (2 pathologists) or malignant (2 pathologists). The accuracy of the case increased from 75% to 100%. As shown in Fig. 3B, C, the small malignant area or scattered malignant tumor cells could be easily missed. After the deep learning algorithm flagged suspicious areas, prompting pathologists to perform a scrutinized reassessment, the accuracies of these two cases increased from 62.5% to 93.75% and 37.5% to 87.5%, respectively.

**Fig. 4:**
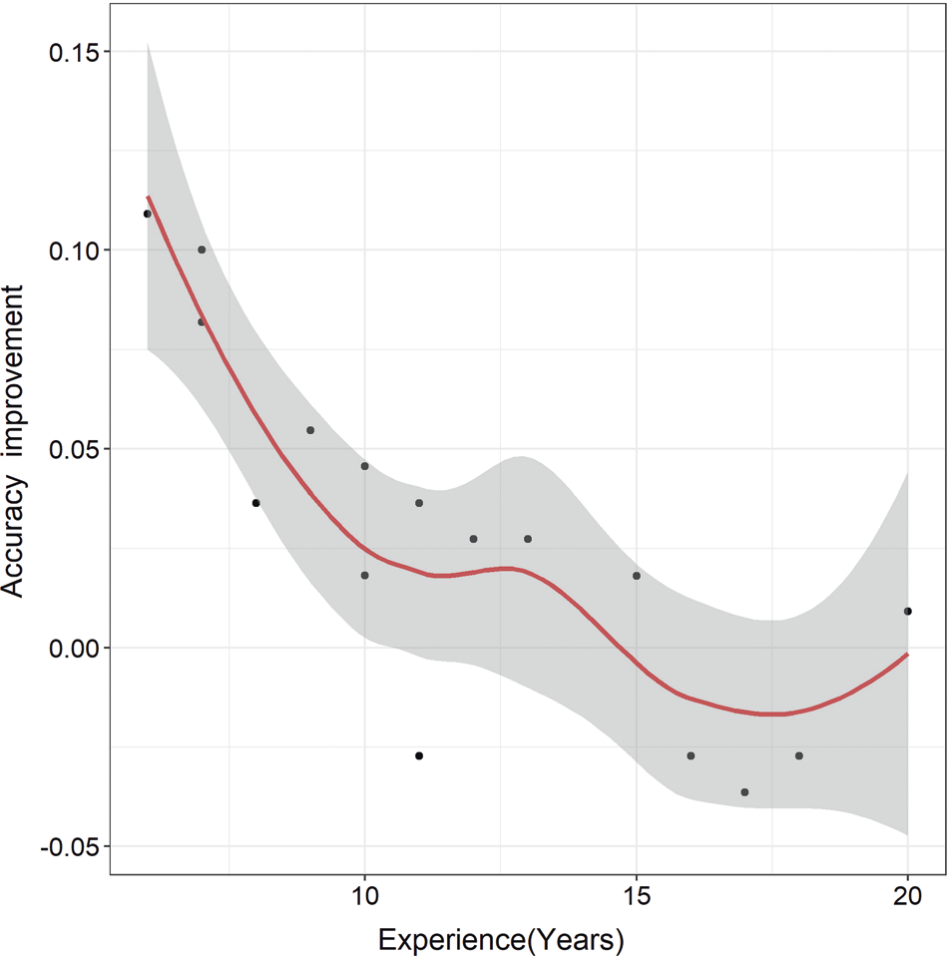
Correlation between deep learning assistance and pathologists’ experiences. Pathologists with less experience tend to obtain larger accuracy improvement from deep learning assistance (the red line representing fitting curve and the shaded area representing 95% confidence intervals).

In addition, we evaluated the correlation between deep learning assistance and pathologists’ experiences. We found that pathologists with less experience tended to obtain larger accuracy improvement from deep learning assistance (Fig. 4).

**Fig. 5:**
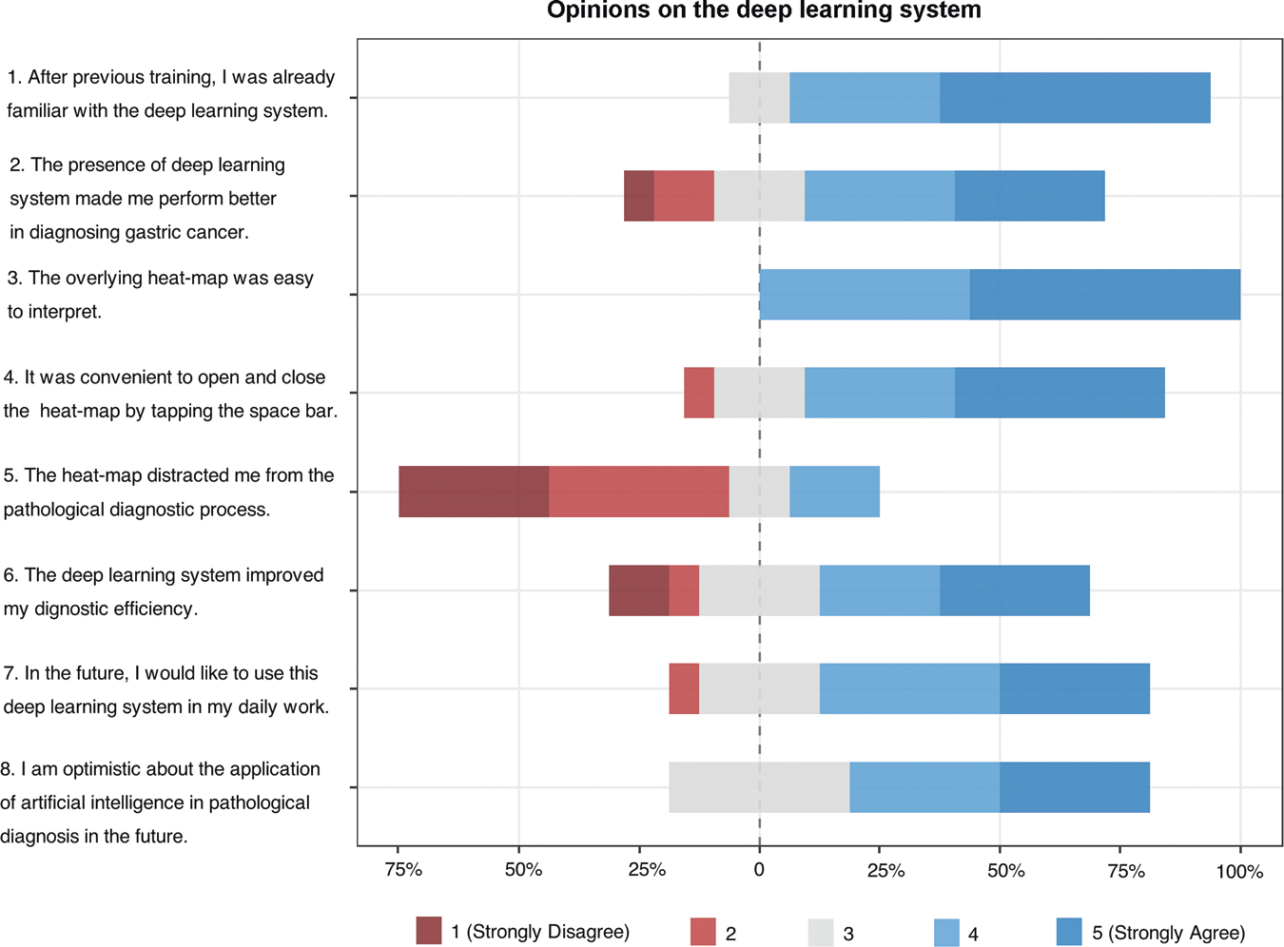
Survey results on the deep learning system. Pathologists were asked to reflect on the pathological diagnosis with and without deep learning assistance and answer questions on a five-point scale from “strongly disagree” to “strongly agree”.

To better understand their perspectives on the deep learning system, we administered a questionnaire to survey the pathologists. The results indicated that most pathologists had an optimistic attitude and were willing to use the deep learning system in their future workflow (Fig. 5).

### WSI review efficiency

The average time of review per WSI for the 16 pathologists without and with deep learning assistance was 26.37 ± 5.22 s (second) and 22.68 ± 4.03 s (*P* = 0.033) (Fig. 2C). We further evaluated the change in review time between different assistance modalities for each pathologist. Deep learning assistance shortened the review time of 12 out of 16 pathologists. The review time saved per WSI varied from 1.2 s to 12.84 s among the 12 pathologists. The detailed results were provided in Supplementary Table S4.

## Discussion

Studies have demonstrated that deep learning could achieve high accuracy in different pathological diagnostic tasks^19–22^. It is notable that deep learning with full automation with no human pathologist backup is not the objective^10,23,24^, and even the best algorithm needs to be integrated into existing clinical workflows to improve patient care. Therefore, we designed a fully crossed MRMC study to investigate the potential of deep learning assistance for pathologists in interpreting digital slides of gastric specimens. Our results demonstrated that deep learning assistance indeed increased the accuracy and efficiency of pathologists in identifying gastric cancer.

In regard to accuracy, we first evaluated the AUC of pathologists with or without deep learning assistance and the results demonstrated that deep learning assistance could improve diagnostic accuracy. Then, we evaluated the sensitivity and specificity between two modalities. Deep learning assistance significantly improved the sensitivity of gastric cancer detection but not specificity. The algorithm implemented in this assessment study has achieved a sensitivity near 100% and a specificity of 80.6% on 3212 real-world WSIs^17^. Algorithm achieving a high sensitivity is often at the cost of decreasing specificity^25,26^. This may be the main reason that deep learning assistance did not improve the specificity of pathologists. In the pathological diagnosis of gastric WSIs, failing to diagnose (a false-negative result) is more harmful than making a gastric cancer (a false-positive result) when it was not. In the clinical workflow, pathologists understand the implications of false positive and false negative for patients, allowing them to optimize the diagnostic operating point and generate different probability heat-maps to fulfill clinical needs, sometimes even on a case-by-case basis.

We further analyzed the change in accuracy for each WSI between assistance modalities. For cases with uncertain diagnoses or small malignant areas, deep learning could significantly improve the diagnostic accuracy. These kinds of situations often occur when pathologists read a slide in haste, such as overloaded with work or the last slide of the day. Deep learning as an analog to a second opinion from a fellow pathologist could not only locate the malignant areas but also provide a malignant probability for each pixel, alerting pathologists to re-scrutinize the potential regions.

As indicated in Fig. 4, pathologists with less experience tend to obtain larger accuracy improvement from deep learning assistance. An implication of this is that pathologists with less experience may have lower confidence in their initial diagnosis, therefore, be more likely to revise the initial diagnosis if it was not in agreement with the prediction of the deep learning algorithm.

Although deep learning assistance significantly improved the average sensitivity of the pathologists, it was still below the ROC of the algorithm, as shown in Fig. 2B. The result implied that the combination of pathologists and deep learning algorithm did not necessarily exceed the algorithm alone. This is mainly because we provided the pathologists only with the heat-map, not a specific slide-level probability, which generates the ROC. Pathologists may selectively believe the predicted malignancy (heat-map) based on their own experience. Although inter- and intra-observer experience variability exists in the pathological diagnosis^27,28^, our results demonstrate that deep learning assistance leads to more reliable and consistent diagnoses, which may result in better treatment decisions.

In addition to accuracy improvement, deep learning assistance also had a time-saving benefit. Although the average review time per WSI was only reduced by <4 s, the cumulative effect may be more notable given the large number of slides in clinical practice. In addition, the 110 WSIs assessed by pathologists were all biopsies, while surgical specimens would greatly prolong pathologists’ review time but not that of the deep learning algorithm. We hypothesize that this time benefit for surgical specimens may be more pronounced. Although 12 out of 16 pathologists spent less reviewing time with deep learning assistance than without, there were 4 pathologists whose review time was prolonged. They generally reflected that the heat-map distracted them from the pathological diagnosing process (Fig. 5). Previous studies have shown that the efficiency gains with deep learning will improve with increased digital pathology experience^29,30^. It is possible that pathologists would spend less time once they get accustomed to the viewer interface of the deep learning system. The time efficiency benefit decreases the workload of pathologists and allows them to spend more time on difficult cases.

There are also several limitations in our study, mainly stemming from the assessment study being performed as a simulation process rather than an actual pathological workflow. The test dataset that the pathologists evaluated with or without deep learning assistance was enriched with cases of gastric cancer, which was not directly comparable to the mixed cases encountered during clinical practice. In our study, each pathologist was given one WSI per case to make the diagnosis. In a real clinical setting, pathologists could access additional slides, additional IHC staining or clinical data to make a final diagnosis. Differences in the inherent difficulty of the assessment set will directly affect the diagnostic performance of pathologists with or without assistance. The algorithm used in this study only detects malignant from benign, without the ability to identify the pathological subtype of gastric cancer, which is related to clinical management and prognosis. Our future research will focus on the establishment of pathological subtype classification, making the system more clinically applicable.

In summary, our study demonstrates that the combination of deep learning and human pathologists has the potential to improve accuracy and efficiency in gastric cancer diagnosis. This research is a useful attempt to understand how deep learning improves pathologists’ diagnosis. Therefore, it further boosts the pathologists' acceptance of this new technique.

## Supplementary information

supplement

## Data Availability

The WSI dataset described in the manuscript were subject to hospital regulations and could not be made available in totality. We provided 50 WSIs and corresponding heat-maps at http://github.com/ThoroughImages/ClinicalPath.

## Code availability

The R code for data visualization was open-sourced at http://github.com/ThoroughImages/ClinicalPath. Meanwhile, we open-sourced the core components of the deep learning system at http://github.com/ThoroughImages/PathologyGo.
