# Supplementary material for: Assessment of deep learning assistance for the pathological diagnosis of gastric cancer
Source: Mod Pathol. 2022 Apr 8;35(9):1262–8. doi: 10.1038/s41379-022-01073-z (PMC9424110; doi:10.1038/s41379-022-01073-z)
Supplement: Supplementary file 1 — supplement [file 41379_2022_1073_MOESM1_ESM.docx]

**Supplementary Materials for**

**Assessment of deep learning assistance for the pathological diagnosis**

**of gastric cancer**

Ba et al.

**
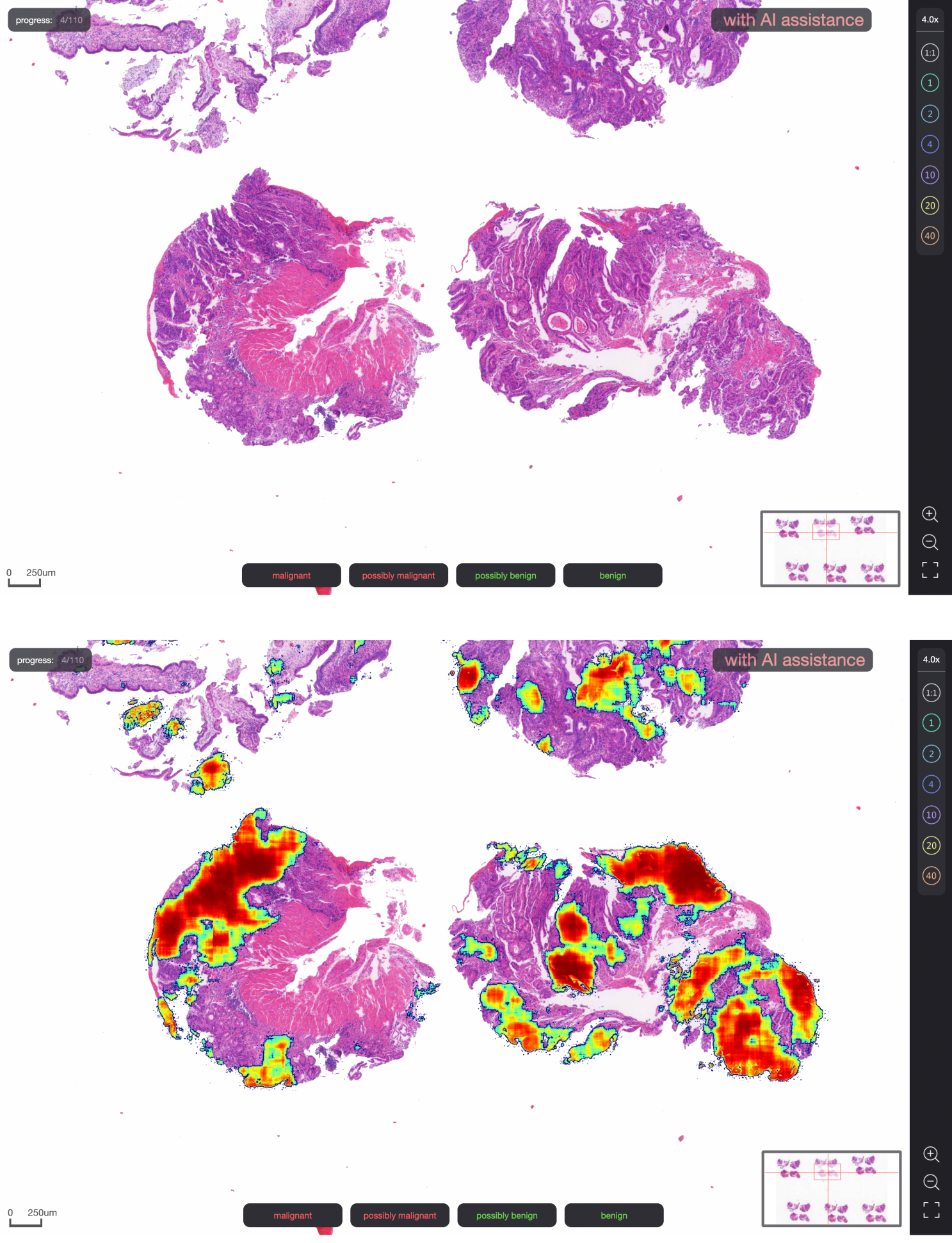
**

**Fig. S1** The interface of the AI assistance system used in assessment study. For WSI with assistance, a heat-map flagging suspicious malignant areas over the WSI can be turned on and off by tapping the space bar on the keyboard (top and bottom). The participants gave the diagnosis by clicking the buttons (malignant/possibly malignant/possibly benign/benign) on the screen.

**Table S1. Each pathologist AUC with or without deep learning assistance**

| Pathologists | AUC with assistance | AUC without assistance | D-value | D-value 95%CI |
| --- | --- | --- | --- | --- |
| Reader 1 | 0.9528 | 0.8908 | 0.0620 | -0.0048,0.1288 |
| Reader 2 | 0.9077 | 0.8320 | 0.0757 | 0.0037,0.1476 |
| Reader 3 | 0.8797 | 0.8995 | -0.0198 | -0.0939,0.0542 |
| Reader 4 | 0.8960 | 0.7465 | 0.1495 | 0.0478,0.2512 |
| Reader 5 | 0.9060 | 0.8600 | 0.0460 | -0.0222,0.1142 |
| Reader 6 | 0.9533 | 0.9495 | 0.0038 | -0.0344,0.0421 |
| Reader 7 | 0.8873 | 0.7467 | 0.1407 | 0.0469,0.2344 |
| Reader 8 | 0.9668 | 0.9320 | 0.0348 | -0.0169,0.0866 |
| Reader 9 | 0.9040 | 0.8190 | 0.0850 | 0.0137,0.1563 |
| Reader 10 | 0.8413 | 0.8118 | 0.0295 | -0.0372,0.0962 |
| Reader 11 | 0.8993 | 0.8895 | 0.0098 | -0.0737,0.0934 |
| Reader 12 | 0.9297 | 0.9575 | -0.0278 | -0.0831, 0.0275 |
| Reader 13 | 0.8707 | 0.7753 | 0.0953 | 0.0105, 0.1802 |
| Reader 14 | 0.9353 | 0.9530 | 0.0177 | -0.0619,0.0265 |
| Reader 15 | 0.9670 | 0.9240 | 0.0430 | -0.0121,0.0981 |
| Reader 16 | 0.8820 | 0.8223 | 0.0597 | -0.0180,0.1373 |
| Average | 0.9112 | 0.8631 | 0.0481 | 0.0176, 0.0786 |

AUC, Area under the receiver operating characteristic curve; D-value (difference value) is defined as AUC with deep leaning assistance minus AUC without; 95 %CI, 95% confidence interval.

**Table S2. Each pathologist sensitivity with or without deep learning assistance**

| Pathologists | Sensitivity with assistance | Sensitivity without assistance | D-value | D-value 95%CI |
| --- | --- | --- | --- | --- |
| Reader 1 | 0.88 | 0.78 | 0.10 | 0.0160, 0.1840 |
| Reader 2 | 0.96 | 0.84 | 0.12 | 0.0290, 0.2110 |
| Reader 3 | 0.92 | 0.98 | -0.06 | -0.1469, 0.0269 |
| Reader 4 | 0.94 | 0.66 | 0.28 | 0.1314, 0.4286 |
| Reader 5 | 0.98 | 0.82 | 0.16 | 0.0574, 0.2626 |
| Reader 6 | 0.92 | 0.82 | 0.10 | -0.0010, 0.2010 |
| Reader 7 | 0.84 | 0.78 | 0.06 | -0.0818, 0.2018 |
| Reader 8 | 0.98 | 0.96 | 0.02 | -0.0484, 0.0884 |
| Reader 9 | 0.90 | 0.64 | 0.26 | 0.1250, 0.3950 |
| Reader 10 | 0.92 | 0.96 | -0.04 | -0.0949, 0.0149 |
| Reader 11 | 0.78 | 0.80 | -0.02 | -0.1512, 0.1112 |
| Reader 12 | 1.00 | 0.98 | 0.02 | -0.0192, 0.0592 |
| Reader 13 | 0.78 | 0.58 | 0.20 | 0.0416, 0.3584 |
| Reader 14 | 0.94 | 0.98 | -0.04 | -0.1184, 0.0384 |
| Reader 15 | 1.00 | 0.96 | 0.04 | -0.0149, 0.0949 |
| Reader 16 | 0.76 | 0.70 | 0.06 | -0.0818, 0.2018 |
| Average | 0.9063 | 0.8275 | 0.0788 | 0.0209, 0.1366 |

D-value (difference value) is defined as sensitivity with deep leaning assistance minus sensitivity without; 95 %CI, 95% confidence interval.

**Table S3. Each pathologist specificity with or without deep learning assistance**

| Pathologists | Specificity with assistance | Specificity without assistance | D-value | D-value 95%CI |
| --- | --- | --- | --- | --- |
| Reader 1 | 0.9500 | 0.9333 | 0.0167 | -0.0569, 0.0902 |
| Reader 2 | 0.7333 | 0.7667 | -0.0333 | -0.1563, 0.0896 |
| Reader 3 | 0.7333 | 0.6333 | 0.1000 | -0.0524, 0.2524 |
| Reader 4 | 0.7167 | 0.7667 | -0.0500 | -0.1852, 0.0852 |
| Reader 5 | 0.6000 | 0.8000 | -0.2000 | -0.3382, -0.0618 |
| Reader 6 | 0.8500 | 0.9833 | -0.1333 | -0.2201, -0.0466 |
| Reader 7 | 0.7333 | 0.5833 | 0.1500 | 0.0116, 0.2884 |
| Reader 8 | 0.8167 | 0.7667 | 0.0500 | -0.0480, 0.1480 |
| Reader 9 | 0.7833 | 0.8500 | -0.0667 | -0.1694, 0.0361 |
| Reader 10 | 0.5000 | 0.5167 | -0.0167 | -0.1524, 0.1191 |
| Reader 11 | 0.9333 | 0.8833 | 0.0500 | -0.0480, 0.1480 |
| Reader 12 | 0.8167 | 0.8500 | -0.0333 | -0.1261, 0.0595 |
| Reader 13 | 0.8500 | 0.9333 | -0.0833 | -0.1798, 0.0132 |
| Reader 14 | 0.8000 | 0.8167 | -0.0167 | -0.1258, 0.0925 |
| Reader 15 | 0.8000 | 0.8000 | 0.0000 | -0.1141, 0.1141 |
| Reader 16 | 0.9000 | 0.9000 | 0.0000 | -0.0807, 0.0807 |
| Average | 0.7823 | 0.7990 | -0.0166 | -0.0637, 0.0304 |

D-value (difference value) is defined as specificity with deep leaning assistance minus specificity without; 95 %CI, 95% confidence interval.

**Table S4. Average review time per WSI for each pathologist with or without deep learning assistance**

| Pathologists | Average review time with assistance (mean ± SD) | Average review time without assistance (mean ± SD) | D-value |
| --- | --- | --- | --- |
| Reader 1 | 18.01±13.71 | 26.87±19.02 | -8.86±25.82 |
| Reader 2 | 26.04±16.92 | 27.96±18.59 | -1.93±21.51 |
| Reader 3 | 20.47±21.08 | 26.78±20.50 | -6.31±26.42 |
| Reader 4 | 18.88±14.24 | 22.17±17.62 | -3.29±23.21 |
| Reader 5 | 17.15±15.01 | 27.56±21.48 | -10.42±28.48 |
| Reader 6 | 23.86±24.88 | 36.70±38.03 | -12.84±45.07 |
| Reader 7 | 22.35±14.79 | 28.68±14.40 | -6.34±19.73 |
| Reader 8 | 22.36±15.58 | 34.46±22.75 | -12.10±21.84 |
| Reader 9 | 24.98±23.82 | 24.33±18.87 | 0.65±29.59 |
| Reader 10 | 28.27±33.88 | 22.92±16.98 | 5.35±37.55 |
| Reader 11 | 20.65±17.68 | 18.38±15.24 | 2.27±18.29 |
| Reader 12 | 28.20±21.93 | 32.67±26.60 | -4.47±28.50 |
| Reader 13 | 28.15±31.80 | 22.13±18.51 | 6.02±37.15 |
| Reader 14 | 16.45±12.43 | 19.75±13.58 | -3.30±16.20 |
| Reader 15 | 26.52±24.17 | 28.79±21.11 | -2.27±28.03 |
| Reader 16 | 20.56±16.50 | 21.76±17.76 | -1.20±21.19 |
| Average | 22.68±4.03 | 26.37±5.22 | -3.69±5.67 |

D-value (difference value) is defined as average review time with deep leaning assistance minus without; SD, standard deviation.
